# Supplementary figures and images for: Residual-aided CSI-free end-to-end learning for multiuser MIMO
Source: PLoS One. 2026 Apr 24;21(4):e0344696. doi: 10.1371/journal.pone.0344696 (PMC13108817; doi:10.1371/journal.pone.0344696)

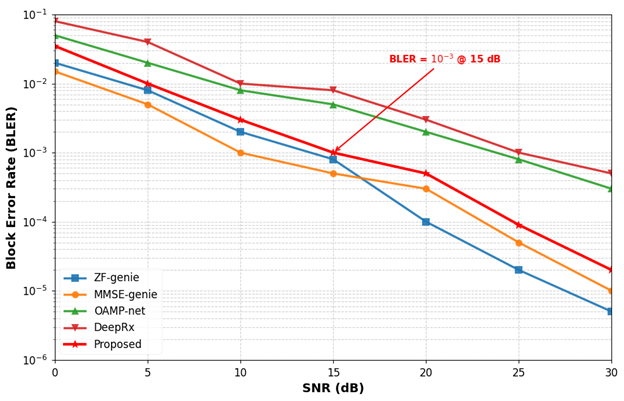

Supplement: S1 Fig — Comprehensive block error rate performance comparison for Rayleigh, Rician, and 3GPP UMi channel models across all examined SNR values. (TIFF) [file pone.0344696.s001.tiff]

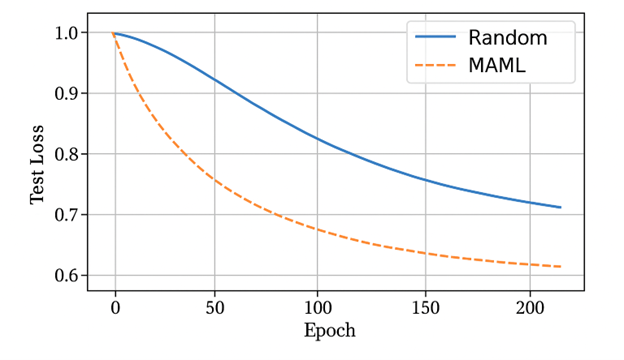

Supplement: S2 Fig — Learning curves for the suggested framework with and without curriculum learning and meta-learning initialisation that demonstrate loss convergence. (TIFF) [file pone.0344696.s002.tiff]

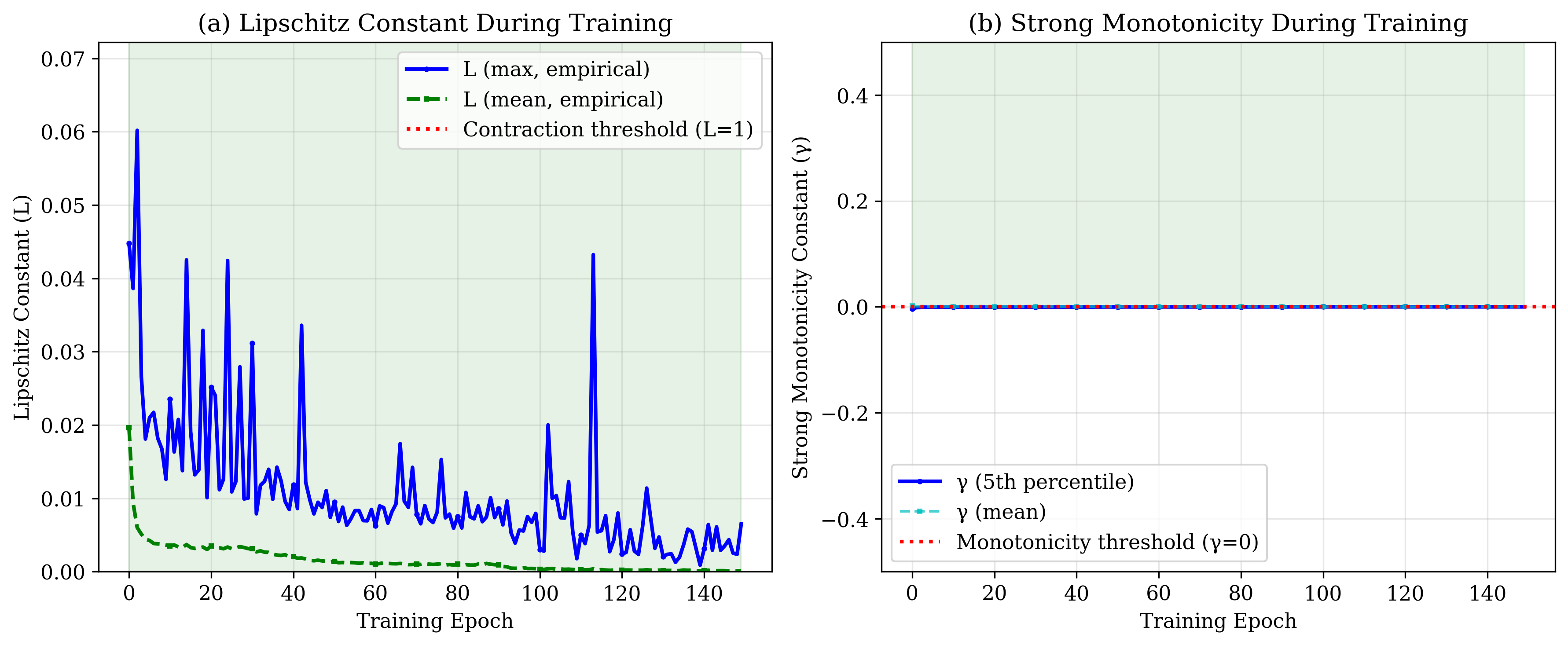

Supplement: S3 Fig — (a) The evolution of the residual mapping Gθ’s empirical Lipschitz constant L throughout training epochs, demonstrating that spectral normalisation keeps L < 1 during optimisation. At initialisation, the Lipschitz constant is roughly 0.02; at convergence, it is less than 0.01. (b) The strong monotonicity constant γ evolves. As mentioned in Remark 2, the network has evolved a quasi-one-shot estimate approach where both L and γ are almost zero. (TIFF) [file pone.0344696.s004.tiff]
