# Supplementary material for: Residual-aided CSI-free end-to-end learning for multiuser MIMO
Source: PLoS One. 2026 Apr 24;21(4):e0344696. doi: 10.1371/journal.pone.0344696 (PMC13108817; doi:10.1371/journal.pone.0344696)
Supplement: S1 Table — All training setups, channel model parameters, and neural network hyperparameters used in the studies are fully specified. (PDF) [file pone.0344696.s003.pdf]

Table 1: **S1 Table. Detailed hyperparameter settings.** Complete specification of neural network hyperparameters, training configuration, and channel model parameters used in all experiments.

(A) Neural Network Architecture

**Component**	**Parameter**	**Value**
4*User Encoder	Input dimension	$L = 4$ bits
	CNN feature maps	128
	CNN kernel size	3
	Output dimension	2 (complex symbol)
6*Graph Transformer	Number of heads	8
	Hidden dimension	256
	Feed-forward dimension	512
	Dropout rate	0.1
	Number of layers	4
	k-NN neighbours (k)	8
4*Residual Refinement	Number of iterations (T)	6
	MLP hidden dimension	256
	Relaxation factor range	$(0.1, 1.9)$
	Spectral normalisation	Enabled

(B) Training Configuration

**Parameter**	**Value**
Optimiser	AdamW
Initial learning rate	10^{-3}
Final learning rate	10^{-5}
Learning rate schedule	Cosine annealing
Batch size	256
Number of epochs	500
Weight decay (λ)	10^{-5}
Gradient clipping	1.0
MI regulariser weight (α)	0.1
Curriculum Learning	
Phase 1 (epochs 1–100)	SNR $\in [15, 25]$ dB
Phase 2 (epochs 101–250)	SNR $\in [5, 25]$ dB
Phase 3 (epochs 251–500)	SNR $\in [0, 30]$ dB
Meta-Learning (MAML)	
Inner loop learning rate	10^{-2}
Inner loop steps	5
Meta batch size	8 tasks

(C) Channel Model Parameters

**Model**	**Parameter**	**Value**
2*Rayleigh	Distribution	$\mathcal{CN}(0, I_N)$
	Coherence time	200 symbols
