# Supplementary material for: Residual-aided CSI-free end-to-end learning for multiuser MIMO
Source: PLoS One. 2026 Apr 24;21(4):e0344696. doi: 10.1371/journal.pone.0344696 (PMC13108817; doi:10.1371/journal.pone.0344696)
Supplement: S2 Table — Detailed FLOP counts for the proposed method and baselines across different user counts (K), loading ratios (β), and graph sparsity levels (k). (PDF) [file pone.0344696.s005.pdf]

Table 1: **S2 Table. Complexity comparison under varying conditions.** Detailed FLOP counts for the proposed DU-SOR method and baselines across different user counts (K), loading ratios ($\beta = K/N$), and graph sparsity levels (k).

(A) FLOPs vs. Number of Users ($N = 128$, $k = 8$)

**Method**	$K = 8$	$K = 16$	$K = 32$	$K = 64$	**Scaling**
MMSE	0.89×10^6	4.2×10^6	24.1×10^6	156.8×10^6	$\mathcal{O}(K^3)$
OAMP-Net	8.4×10^6	18.2×10^6	42.5×10^6	68.7×10^6	$\mathcal{O}(K^2)$
GNN-Det	12.1×10^6	28.4×10^6	58.2×10^6	89.2×10^6	$\mathcal{O}(K^2)$
DeepRx	38.5×10^6	40.2×10^6	41.8×10^6	42.3×10^6	$\mathcal{O}(1)^*$
**Proposed**	$\mathbf{1.8 \times 10^6}$	$\mathbf{2.9 \times 10^6}$	$\mathbf{4.2 \times 10^6}$	$\mathbf{5.8 \times 10^6}$	$\mathcal{O}(K \log K)$

*DeepRx uses fixed-size CNN; complexity independent of K but high constant factor.

(B) FLOPs vs. Loading Ratio ($K = 32$)

**Method**	$\boldsymbol{\beta = 0.125}$ ($N = 256$)	$\boldsymbol{\beta = 0.25}$ ($N = 128$)	$\boldsymbol{\beta = 0.5}$ ($N = 64$)	$\boldsymbol{\beta = 1.0}$ ($N = 32$)
MMSE	26.8×10^6	24.1×10^6	22.4×10^6	21.5×10^6
GNN-Det (full)	124.5×10^6	58.2×10^6	28.4×10^6	14.2×10^6
**Proposed**	$\mathbf{6.8 \times 10^6}$	$\mathbf{4.2 \times 10^6}$	$\mathbf{2.8 \times 10^6}$	$\mathbf{1.9 \times 10^6}$

(C) Effect of Graph Sparsity ($K = 32$, $N = 128$)

**Sparsity (k)**	**4**	**8**	**16**	**32**	**Full (N)**
FLOPs ($\times 10^6$)	2.8	4.2	6.1	9.8	58.2
VRAM (GB)	1.4	2.1	2.9	3.8	4.5
BLER @ 15dB	1.18×10^{-3}	1.02×10^{-3}	0.98×10^{-3}	0.96×10^{-3}	0.95×10^{-3}
Relative Perf.	0.85	1.00	1.02	1.03	1.04

(D) Theoretical vs. Measured Complexity

**Users (K)**	**Theoretical**	**Measured**	**Ratio**	**Scaling Factor**
8	1.66×10^6	1.82×10^6	1.10	–
16	2.77×10^6	2.94×10^6	1.06	1.62
32	4.43×10^6	4.21×10^6	0.95	1.43
64	6.64×10^6	5.83×10^6	0.88	1.38

Theoretical: $c \cdot K \log_2 K$ with $c = 6.9 \times 10^4$. Scaling factor: FLOPs($2K$)/FLOPs(K).
For $\mathcal{O}(K \log K)$: expected ratio ≈ 2.2. For $\mathcal{O}(K^2)$: expected ratio $= 4$.
