# Supplementary material for: Residual-aided CSI-free end-to-end learning for multiuser MIMO
Source: PLoS One. 2026 Apr 24;21(4):e0344696. doi: 10.1371/journal.pone.0344696 (PMC13108817; doi:10.1371/journal.pone.0344696)
Supplement: Attachment — Submitted filename: Response_to_Reviewers.pdf [file pone.0344696.s007.pdf]

Response to Reviewers

Manuscript ID: PONE-D-25-64863
Title: Residual-Aided CSI-free End-to-End Learning for Multiuser MIMO

We sincerely thank the Academic Editor and Reviewers for their constructive feedback. We have carefully addressed each comment, and the manuscript has been substantially improved. Below we provide point-by-point responses.

Reviewer #1

Reviewer Comment: *1. The novelty is not clearly established, as the proposed framework mainly combines existing techniques without clearly identifying a fundamentally new contribution.*

Response: We agree that the novelty statement required clarification. The unique contribution lies in the **synergistic integration** of sparse graph transformers with iterative residual refinement for **CSI-free** detection—a combination not previously explored. Unlike OAMP-Net (requires CSI) or DeepRx (black-box CNN), our DU-SOR uses graph priors to condition blind residual updates.

Change Made: Added explicit numbered contribution list in Introduction. Added "Distinction from Prior Work" paragraph clarifying differences from OAMP-Net, DeepRx, and recent GNNs. Renamed framework to "DU-SOR" throughout.

Reviewer Comment: *2. Several key claims are overstated, especially the assertion that residual learning alone leads to a complexity reduction from O(K^3) to O(K log K).*

Response: We have corrected this. The complexity reduction is attributed to the **sparse attention mechanism**, not residual learning alone. Residual connections improve convergence and gradient flow; the complexity gain comes from limiting attention to $k \approx \log N$ neighbours.

Change Made: Added **Proposition 1** with formal proof attributing complexity to sparse adjacency matrix. Revised all claims to correctly credit sparse attention.

Reviewer Comment: *3. The paper lacks theoretical support, with no convergence, stability, or performance analysis explaining why CSI-free detection can approach MMSE-genie bounds.*

Response: We have added rigorous theoretical analysis.

Change Made: Added **Theorem 1** (Convergence of Residual Refinement) based on Banach Fixed Point Theorem with complete proof. Added explanation of spectral normalisation for Lipschitz constraint enforcement.

Reviewer Comment: *4. Important implementation details are unclear, particularly the graph construction, attention sparsification, and how the claimed complexity scaling is practically achieved.*

Response: We have significantly expanded implementation details.

Change Made: Added **Algorithm 1** with complete pseudocode. Added explicit description of k-NN graph construction. Clarified sparse mask A in attention equation (Eq. 5). Added proof showing how $k \approx \log N$ leads to $O(K \log K)$ complexity.

Reviewer Comment: *5. The experimental evaluation is limited to simulations, with relatively weak baselines and insufficient justification for some reported metrics (e.g., energy and power results).*

Response: We acknowledge the simulation limitation and have strengthened baselines and metrics.

Change Made: Added comparison with post-2023 GNN-Detector [39] in Table 2. Added OAMP-Net column. Specified that power was measured using **NVML polling at 10ms intervals** rather than TDP estimates. Added statement that all baselines were retrained on identical dataset. Added explicit Limitations subsection acknowledging simulation-only evaluation.

Reviewer #2

Reviewer Comment: *1. Supplement comparisons with similar Graph Transformer works post-2023 to strengthen advantages.*

Response: Added comparisons with Lau et al. (Neurocomputing, 2024) and Wang et al. (IEEE TWC, 2023).

Change Made: New citations [39], [40] added. Table 2 now includes GNN-Detector (2024) column showing 18.2ms latency, 4.5GB VRAM, 235W power vs. our 8.6ms, 2.1GB, 150W.

Reviewer Comment: *2. Experiments cover various simulated channel models but lack test data in real propagation environments.*

Response: We acknowledge this limitation. Real-world validation with SDR testbeds is proposed as future work.

Change Made: Added explicit statement in Limitations subsection. Added "Field Trials" as Future Direction item 3.

Reviewer Comment: *3. Supplement hardware resource consumption data to support deployment evaluation.*

Response: Hardware metrics are now comprehensively reported.

Change Made: Added **Table 2** with Inference Latency, VRAM Usage, Peak Power (NVML-measured), and FLOPs for all methods. Added ablation showing VRAM increase to 4.2GB without sparse attention.

Reviewer Comment: *4. Currently, only single-base-station uplink scenarios are verified. It is recommended to extend to typical scenarios to broaden the framework's application coverage.*

Response: Multi-BS extension is proposed as future work.

Change Made: Added "Distributed MIMO" as Future Direction item 2, proposing extension to CoMP and Cell-Free Massive MIMO where adjacency matrix represents inter-BS connectivity.

Reviewer Comment: *5. Explore lightweight strategies to reduce resource thresholds for training and deployment.*

Response: Lightweight strategies are proposed as future work.

Change Made: Added "Lightweight Strategies" as Future Direction item 1, proposing INT8 quantisation and structured pruning for edge deployment.

Journal Requirements

Reviewer Comment: *1. Please ensure that your manuscript meets PLOS ONE's style requirements.*

Response: We have verified compliance with PLOS ONE formatting templates.

Change Made: Verified title capitalisation, author affiliations format, figure caption format, and reference style against PLOS ONE templates.

Reviewer Comment: *2. PLOS ONE has specific guidelines on code sharing.*
Response: Code and data will be made fully available.
Change Made: Added Data Availability Statement with GitHub repository URL and Zenodo DOI placeholder.

Reviewer Comment: *3. When completing the data availability statement, you indicated you will make your data available on acceptance.*
Response: Data availability statement has been updated.
Change Made: Statement now reads: "All data underlying the findings in this study, along with the source code required to reproduce the results, are fully available without restriction." Repository links provided.

Summary of Major Changes

1. Added formal theoretical analysis: Theorem 1 (convergence) and Proposition 1 (complexity)
2. Added Algorithm 1 with complete pseudocode
3. Added Table 2 with measured hardware metrics (NVML)
4. Added post-2023 GNN baseline comparisons [39], [40]
5. Added explicit Contributions list and "Distinction from Prior Work" paragraph
6. Added Limitations and Future Directions subsections
7. Added Data Availability Statement
8. Corrected complexity claims to attribute scaling to sparse attention
