# Supplementary material for: Residual-aided CSI-free end-to-end learning for multiuser MIMO
Source: PLoS One. 2026 Apr 24;21(4):e0344696. doi: 10.1371/journal.pone.0344696 (PMC13108817; doi:10.1371/journal.pone.0344696)
Supplement: Attachment — Submitted filename: Response_to_Reviewers_auresp_2.pdf [file pone.0344696.s008.pdf]

Response to Reviewers

Manuscript ID: PONE-D-25-64863R1
Title: Residual-Aided CSI-free End-to-End Learning for Multiuser MIMO: A Deep Unfolding Approach

We sincerely thank the Academic Editor and Reviewers for their constructive feedback. We have carefully addressed each comment, and the manuscript has been substantially improved. Below we provide point-by-point responses.

Reviewer #1

Reviewer Comment: *1. The link between the proposed DU-SOR framework and classical Successive Over-Relaxation (SOR) is unclear. The update rules mainly resemble residual refinement, without an explicit relaxation factor or clear correspondence to standard SOR iterations. The authors should clarify this connection or reconsider the terminology.*

Response: We agree that the connection to classical SOR required clarification. The classical SOR method for solving linear systems $Ax = b$ has the form $x^{(t+1)} = x^{(t)} + \omega(D+\omega L)^{-1}r^{(t)}$ where $\omega \in (0, 2)$ is the relaxation parameter. Our framework generalises this by: (i) replacing the fixed linear operator $(D + \omega L)^{-1}$ with a learned nonlinear mapping $G_\theta^{(t)}$, and (ii) making the relaxation factor data-adaptive via $\omega^{(t)} = \sigma(MLP_\omega(Z^{(t)}))$ where $\sigma(\cdot)$ is a sigmoid scaled to $(0, 2)$.

Change Made: Added dedicated "Connection to Classical SOR" paragraph in Section "Materials and Methods" (page 7). Added Equation 7 showing classical SOR form and Equation 8 showing our learned generalisation. Explicitly stated how the sigmoid scaling to $(0, 2)$ matches the classical SOR convergence range.

Reviewer Comment: *2. The convergence analysis based on the Banach fixed-point theorem is not fully convincing. While spectral normalization is mentioned, it is not rigorously shown that the overall network mapping is strictly contractive, nor is it explained why convergence implies near-MMSE performance.*

Response: We have completely revised the convergence analysis with a mathematically rigorous proof. The previous proof incorrectly used the triangle inequality, yielding a factor $(1+\omega L_G) > 1$ which cannot establish contraction. The corrected proof uses squared-norm expansion and explicitly requires two conditions: (1) Lipschitz continuity with constant L_G, and (2) strong monotonicity with constant $\gamma > 0$, such that $\langle G_\theta(u, y) - G_\theta(v, y), u - v\rangle \leq -\gamma\|u - v\|^2$. Under these conditions and $\omega < 2\gamma/L_G^2$, we derive the contraction factor $q^2 = 1 - 2\omega\gamma + (\omega L_G)^2 < 1$.

Change Made: Completely rewrote **Theorem 1** (Convergence of Residual Refinement) with corrected proof using squared-norm expansion. Added explicit assumptions (Lipschitz continuity and strong monotonicity) in the theorem statement. Added **Remark** explaining that strong monotonicity reflects training to approximate a descent direction, and that Lipschitz continuity is enforced via spectral normalisation. Added reference to S3 Fig showing empirical verification that Lipschitz constant remains below 1 throughout training.

Reviewer Comment: *3. The claimed complexity reduction from $O(K^3)$ to $O(K \log K)$ relies on strong assumptions that are insufficiently justified, particularly regarding the relationship between the number of graph nodes and the number of users in massive MIMO settings.*

Response: We have added detailed justification for the complexity claims. The graph $\mathcal{G} = (\mathcal{V}, \mathcal{E})$ is constructed with $|\mathcal{V}| = N$ nodes corresponding to BS antenna elements. In massive MIMO

systems with loading ratio $\beta = K/N$ (typically $\beta \in [0.1, 0.5]$), both K and N scale together. The sparse k-NN attention with $k = c \log N$ neighbours yields $O(N \log N)$ complexity per iteration, which translates to $O(K \log K)$ under fixed β.

Change Made: Added "Graph-System Mapping" paragraph explicitly clarifying the relationship between graph nodes (N) and users (K). Added three-step proof in **Proposition 1** showing: (Step 1) sparse attention complexity, (Step 2) justification for $k = c \log N$ based on spectral properties of random geometric graphs and antenna coupling decay, (Step 3) complexity derivation. Added "Conditions and Limitations" paragraph stating when the bound holds and acknowledging worst-case $O(K^2)$ for highly correlated channels.

Reviewer Comment: *4. Important implementation details affecting reproducibility are missing, including the definition of features used for k-NN graph construction, the distance metric, and whether the graph is dynamically constructed per sample.*

Response: We have significantly expanded the implementation details to ensure full reproducibility.

Change Made: Added complete "Graph construction details" subsection specifying:

- **Node Features:** $f_n = [|y_n|, \angle y_n, p_n^x, p_n^y] \in R^4$ where $|y_n|$ and $\angle y_n$ are magnitude and phase of received signal, and (p_n^x, p_n^y) are normalised antenna coordinates (Equation 9).
- **Distance Metric:** Euclidean distance $d(v_i, v_j) = \|f_i - f_j\|_2$ (Equation 10).
- **Graph Sparsity:** $k = 8$ nearest neighbours based on sensitivity analysis reported in Ablation Studies.
- **Static vs. Dynamic:** Hybrid approach where base topology is precomputed from antenna geometry (static) while edge weights are dynamically updated each forward pass. Formal adjacency matrix definition with learnable temperature parameter τ (Equation 11).

Reviewer Comment: *5. The "CSI-free" and end-to-end system assumptions require further discussion, especially regarding the practicality of deploying a learnable encoder at the user equipment and its compatibility with standard communication systems.*

Response: We have added a comprehensive discussion of practical deployment considerations addressing encoder complexity, standards compatibility, model distribution, and fallback modes.

Change Made: Added "Practical deployment considerations" subsection in Discussion covering:

- **Encoder Complexity:** ~47,000 parameters, <200 KB storage, <0.5 ms latency on Snapdragon 888.
- **Compatibility with Standards:** Power normalisation layer ensures outputs lie within standard QAM constellation regions, enabling graceful fallback for legacy receivers.
- **Model Distribution:** Three mechanisms—factory provisioning, System Information Block (SIB) broadcast, and federated refinement.
- **Hybrid Deployment Mode:** Decoder-only mode where UEs use standard 64-QAM and only BS employs neural decoder, achieving 70% of full E2E gains with no UE modification.

Reviewer #2

Reviewer Comment: *I have no further comments on the methodology or results. I agree with acceptance of the manuscript. However, formatting issues still remain, particularly regarding punctuation around equations and the reference list. The journal name abbreviations are not consistently*

or correctly formatted. The authors should carefully revise the manuscript by thoroughly checking against recently published papers and applying the journal's formatting rules throughout.

Response: We thank the reviewer for their positive assessment. We have carefully revised all formatting issues throughout the manuscript.

Change Made: Made the following formatting corrections:

- **Equation Punctuation:** Reviewed all 16 equations and ensured proper punctuation (commas when followed by "where" clauses, periods at end of sentences). Fixed adjacency matrix equation (Eq. 11) to include period.
- **Bibliography Format:** Reformatted all 55 references to Vancouver/ICMJE style as required by PLOS ONE:
  - Standardised journal abbreviations (e.g., "IEEE Trans Wirel Commun" consistently)
  - Corrected page number truncation (e.g., 134-42 instead of 134-142)
  - Ensured "et al." used after 6 authors
  - Corrected conference proceedings format with location, date, and publisher
- **Figure References:** Standardised to "Fig" without period throughout.
- **Figure Numbering:** Changed to consecutive numbering (Fig 1, Fig 2, Fig 3).
- **Terminology:** Changed "huge MIMO" to "massive MIMO" (standard terminology).

Summary of Major Changes

1. Added "Connection to Classical SOR" paragraph with explicit equations showing relationship to classical method (Eqs. 7–8)

2. Completely revised **Theorem 1** with mathematically rigorous convergence proof using squared-norm expansion and explicit assumptions

3. Added "Graph-System Mapping" paragraph and three-step proof in **Proposition 1** justifying $O(K \log K)$ complexity

4. Added complete "Graph construction details" subsection with node features, distance metric, sparsity, and static/dynamic construction (Eqs. 9–11)

5. Added "Practical deployment considerations" subsection addressing encoder complexity, standards compatibility, model distribution, and hybrid mode

6. Corrected all equation punctuation throughout manuscript

7. Reformatted entire bibliography to Vancouver/ICMJE style

8. Standardised figure references and numbering

We believe these revisions fully address all reviewer concerns and significantly strengthen the manuscript. We thank the reviewers for their valuable feedback that has improved the quality of this work.

Sincerely,
Emmanuel Ampoma Affum (on behalf of all authors)
